# Investigating potential molecular mechanisms of serum exosomal miRNAs in colorectal cancer based on bioinformatics analysis

**DOI:** 10.1097/MD.0000000000022199

**Published:** 2020-09-11

**Authors:** Haifeng Wang, Xiliang Chen, Lingling Bao, Xuede Zhang

**Affiliations:** a Department of Hematology and Oncology, Beilun District People's Hospital, Ningbo, Zhejiang; b Department of Clinical Laboratory, Zhangqiu District People's Hospital, Jinan, Shandong, China.

**Keywords:** bioinformatics analysis, colorectal cancer, differentially expressed microRNAs, hub gene, serum exosomes

## Abstract

Colorectal cancer (CRC) is the most common malignant gastrointestinal tumor worldwide. Serum exosomal microRNAs (miRNAs) play a critical role in tumor progression and metastasis. However, the underlying molecular mechanisms are poorly understood.

The miRNAs expression profile (GSE39833) was downloaded from Gene Expression Omnibus (GEO) database. GEO2R was applied to screen the differentially expressed miRNAs (DEmiRNAs) between healthy and CRC serum exosome samples. The target genes of DEmiRNAs were predicted by starBase v3.0 online tool. The gene ontology (GO) and Kyoto Encyclopedia of Genomes pathway (KEGG) enrichment analysis were performed using the Database for Annotation, Visualization and Integrated Discovery (DAVID) online tool. The protein-protein interaction (PPI) network was established by the Search Tool for the Retrieval of Interacting Genes (STRING) visualized using Cytoscape software. Molecular Complex Detection (MCODE) and cytohubba plug-in were used to screen hub genes and gene modules.

In total, 102 DEmiRNAs were identified including 67 upregulated and 35 downregulated DEmiRNAs, and 1437 target genes were predicted. GO analysis showed target genes of upregulated DEmiRNAs were significantly enriched in transcription regulation, protein binding, and ubiquitin protein ligase activity. While the target genes of downregulated DEmiRNAs were mainly involved in transcription from RNA polymerase II promoter, SMAD binding, and DNA binding. The KEGG pathway enrichment analyses showed target genes of upregulated DEmiRNAs were significantly enriched in proteoglycans in cancer, microRNAs in cancer, and phosphatidylinositol-3 kinases/Akt (PI3K-Akt) signaling pathway, while target genes of downregulated DEmiRNAs were mainly enriched in transforming growth factor-beta (TGF-beta) signaling pathway and proteoglycans in cancer. The genes of the top 3 modules were mainly enriched in ubiquitin mediated proteolysis, spliceosome, and mRNA surveillance pathway. According to the cytohubba plugin, 37 hub genes were selected, and 4 hub genes including phosphoinositide-3-kinase regulatory subunit 1 (PIK3R1), SRC, cell division cycle 42 (CDC42), E1A binding protein p300 (EP300) were identified by combining 8 ranked methods of cytohubba.

The study provides a comprehensive analysis of exosomal DEmiRNAs and target genes regulatory network in CRC, which can better understand the roles of exosomal miRNAs in the development of CRC. However, these findings require further experimental validation in future studies.

## Abstract 2

Supplemental Digital Content is available in the text

## 1 Introduction

Colorectal cancer (CRC) is one of the most frequent malignancies in the gastrointestinal tract worldwide. Although diagnostic and treatment methods have been progressed over the past decades, CRC is still the second most common cause of cancer mortality.^[1]^ Although the 5-year relative survival rate was above 90% for patients with localized stage colorectal cancer, only about 14% for patients with distant tumor spread.^[2]^ Therefore, early detection of CRC and understanding the molecular mechanism of distant metastases are urgently required. Several studies indicates detection of serum exosomes may be considered a non-invasive diagnosis of cancer and contribute to clarify the potential mechanisms for distant metastasis.^[3–5]^

Exosomes are small vesicles of 40 to 100 nm in diameter and derived from a fusion of multivesicular body (MVB) with plasma membrane.^[6]^ Exosomes are secreted from multiple cell types, including malignant tumor cells and released into the circulation and other body fluids.^[7]^ Exosomes contain proteins, mRNAs, and miRNAs, which could be detected in the human body fluids and participate in intercellular communication.

MiRNAs are short, endogenous, non-coding RNAs involved in post-transcriptional regulation of gene expression.^[8]^ To date, miRNAs have been reported to be involved in many physiological and pathological processes through regulating gene expression by binding to target mRNAs including translation repression or RNA degradation. Aberrant expression profiles of miRNAs are widely found in various cancers and used to explore the underlying molecular mechanism of cancer. Several studies have found miRNAs contained in exosomes are protected from RNase degradation, which may be useful diagnostic biomarkers for cancer detection and disease monitoring.^[9,10]^ Recent studies have indicated exosome-delivered miRNAs played a critical role in carcinogenesis and cancer progression in CRC.^[11]^ For example, miR-210, miR-220, miR-141 have been implicated in regulating epithelial-mesenchymal transition (EMT) of CRC cells.^[12,13]^ MiR-379 may be involved in CRC cell proliferation and migration.^[14]^ MiR-17–92a, miR-193a, let-7a, and miR-150 were promising biomarkers and therapeutic targets for CRC.^[15–17]^ Therefore, a comprehensive understanding of exosomal miRNAs and their mRNA targets may provide promising candidate targets for early diagnosis and therapeutic intervention and be useful to clarify the potential molecular mechanisms of CRC.

In the present study, we downloaded the miRNA expression profile of GSE39833 from the Gene Expression Omnibus (GEO). The GEO2R online tool was used to identify DEmiRNAs in serum exosomes of primary CRC patients and healthy controls. Then, the target genes of DEmiRNAs were predicted using starBase v3.0 online datasets and gene ontology (GO) function, and the Kyoto Encyclopedia of Genomes pathway (KEGG) pathways enrichment analyses were conducted. Finally, the PPI networks were established to investigate sub-module analysis and hub genes related to serum exosomal miRNA in CRC. The study aimed to obtain further insight into the underlying mechanisms and to explore the potential biomarkers and therapeutic targets for CRC.

## 2 Materials and methods

Ethical approval or patient consent was not necessary since the present study was an integrative analysis of published data.

### 2.1 Microarray data.

The serum exosomal miRNA expression profile of GSE39833 was downloaded from the GEO database (http://www.ncbi.nlm.nih.gov/geo/). The GSE39833 dataset based on GPL14767 (Agilent-021827 Human miRNA Microarray G4470C) contained 99 samples, including 11 healthy serum exosome samples and 88 CRC serum exosome samples.^[15]^

### 2.2 Identification of DEmiRNAs

In this study, GEO2R was applied to screen DEmiRNAs between healthy and CRC serum exosome samples. GEO2R (http://www.ncbi.nlm.nih.gov/geo/geo2r/) is an online analysis tool for comparing 2 groups of GEO datasets based on the GEO query and Limma R packages.^[18]^ The miRNAs that met the cut-off criteria of the adjusted *P* value (adj. P) < .05 and |log FC | > 2.0 were considered as DEmiRNAs.

### 2.3 Prediction of DEmiRNAs target genes

The target genes of DEmiRNAs were predicted using starBase v3.0 (http://starbase.sysu.edu.cn/),^[19]^ which is an open-source platform for studying the miRNA-mRNA interactions produced by 7 established prediction databases (microT, miRanda, miRmap, PITA, RNA22, PicTar, and TargetScan). In this study, the genes predicted by PITA, miRmap, microT, miRanda, PicTar, and TargetScan simultaneously were considered as the targets of DEmiRNAs.

### 2.4 GO and KEGG pathway enrichment analysis of target genes of DEmiRNAs

GO analysis is a common useful method for functional enrichment analysis on the high-throughput genome or transcriptome data and GO terms can be classified into the biological process (BP), molecular function (MF), and cellular component (CC).^[20]^ KEGG (http://www.genome.jp/) is a knowledge database for systematic analysis of gene functions, linking genomic information with high-level functions and utilities of the biological system.^[21]^ In this study, GO function and KEGG pathway enrichment analysis for target genes of DEmiRNAs were performed using the Database for Annotation, Visualization and Integrated Discovery (DAVID) online tool. *P* < .05 was considered statistically significant.

### 2.5 Integration of protein–protein interaction (PPI) network and module analysis

The Search Tool for the Retrieval of Interacting Genes (STRING) database (http://string-db.org/) is online tool designed to analyze the PPI information. The target genes predicted by DEmiRNAs were mapped to STRING and only interactions with a combined score >0.9 were selected as significant. Subsequently, the PPI network with significant gene pairs were visualized using cytoscape software. The Molecular Complex Detection (MCODE) plug-in was applied to screen of PPI network in cytoscape software. The criteria were set as follows: degree cutoff = 2, node score cutoff = 0.2, k-core = 2 and max depth = 100. Additionally, the KEGG pathway enrichment analysis was performed for genes in the modules.

### 2.6 Identification of hub gene and construction of the miRNA-mRNA network

Hub genes have many interactions with other genes and usually play an important role in a biological system. The plug-in cytohubba was applied to screen the potential hub genes by several topological algorithms including Degree, Edge Percolated Component (EPC), Maximum Neighborhood Component (MNC), Density of Maximum Neighborhood Component (DMNC), Maximal Clique Centrality (MCC) and centralities based on shortest paths, such as Bottleneck (BN), EcCentricity, Closeness, Radiality, Betweenness, and Stress.^[22]^ All hub genes were sorted according to the 11 centrality parameters to identify highly connected nodes. The top 10 hub genes according to 11 ranked methods and DEmiRNAs that target them were selected to construct a miRNA-mRNA regulatory network using Cytoscape software.

## 3 Results

### 3.1 Identification of DEmiRNAs

In total, 102 DEmiRNAs were identified in the gene expression profile of GSE39833 including 67 upregulated DEmiRNAs and 35 downregulated DEmiRNAs in the CRC compared with normal control, using adjusted *P* value < .05 and |logFC| ≥ 2 as cut-off criteria. The top 10 upregulated and downregulated DEmiRNAs were listed in Table 1 (all of the 102 DEmiRNAs are listed in Supplementary Material 1).

**Table 1:**
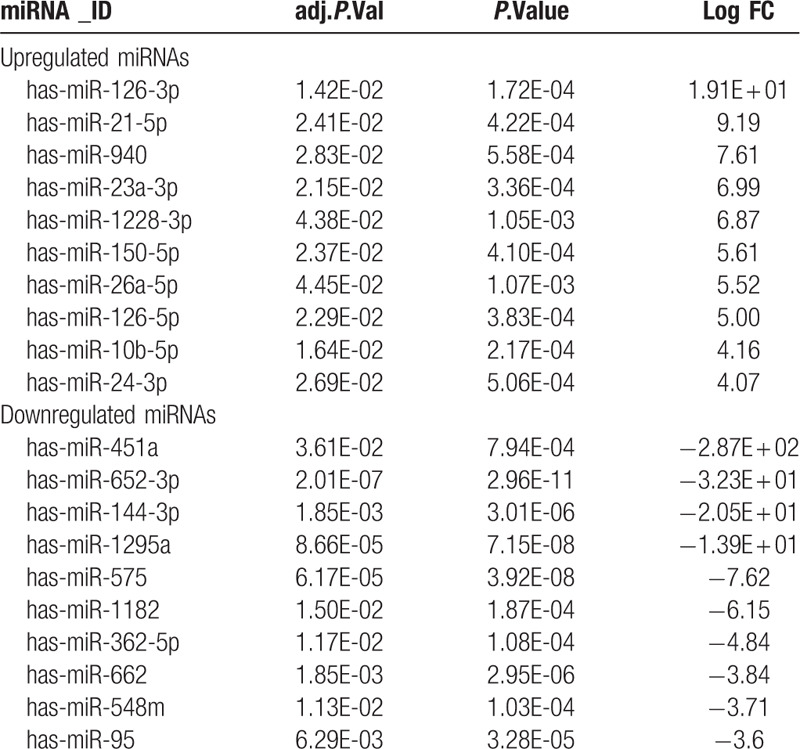
Top 10 most significantly up or downregulated DEmiRNAs in serum exosome.

### 3.2 Identification of DEmiRNAs target genes

To elucidate the function of the DEmiRNAs in CRC, the prediction of their target genes were performed using starBase v3.0 database. In order to identify reliable DEmiRNAs target genes, the genes predicted by all the 6 programs (PITA, miRmap, microT, miRanda, PicTar, and TargetScan) were identified as DEmiRNAs target genes. Finally, a total of 1437 target genes of DEmiRNAs were predicted, including 1095 target genes of upregulated DEmiRNAs and 620 target genes of downregulated DEmiRNAs.

### 3.3 GO analysis of target genes

GO functional analysis of DEmiRNAs target genes was conducted using the online software DAVID. In BP group, the target genes of upregulated DEmiRNAs were mainly enriched in “positive regulation of transcription from RNA polymerase II promoter”, “positive regulation of transcription, DNA-templated”, “negative regulation of transcription from RNA polymerase II promoter”, “transcription from RNA polymerase II promoter”, and “negative regulation of transcription, DNA-templated”. The target genes of downregulated DEmiRNAs were primarily enriched in “positive regulation of transcription from RNA polymerase II promoter”, “transcription from RNA polymerase II promoter”, “positive regulation of transcription, DNA-templated”, “transcription, DNA-templated” and “negative regulation of transcription from RNA polymerase II promoter”. In MF group, the target genes of upregulated DEmiRNAs were mainly enriched in “protein binding”, “transcription factor activity, sequence-specific DNA binding”, “ubiquitin protein ligase activity”, “transcriptional activator activity, RNA polymerase II core promoter proximal region sequence-specific binding” and “sequence-specific DNA binding”. The target genes of downregulated DEmiRNAs were mostly enriched in “protein binding”, “transcription factor activity, sequence-specific DNA binding”, “sequence-specific DNA binding”, “SMAD binding”, and “DNA binding”. In CC group, the target genes of upregulated DEmiRNAs were mainly enriched in “nucleus”, “nucleoplasm”, “cytoplasm”, “membrane”, and “cytosol”. The target genes of downregulated DEmiRNAs were primarily enriched in “nucleoplasm”, “nucleus”, “cytoplasm”, “cytosol”, and “growth cone” (Table 2).

**Table 2:**
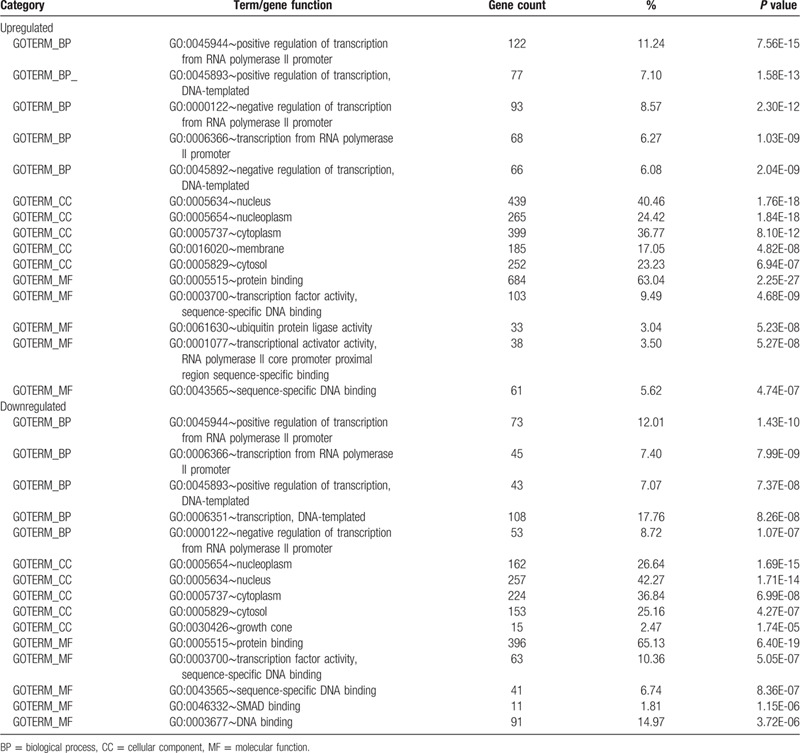
Gene ontology analysis of DEmiRNAs target genes (Top 5).

### 3.4 KEGG pathway enrichment analysis of target genes of DEmiRNAs

KEGG pathway enrichment analysis revealed that the target genes of upregulated DEmiRNAs were significantly enriched in proteoglycans in cancer, microRNAs in cancer, phosphatidylinositol-3 kinases/Akt (PI3K-Akt) signaling pathway, The forkhead box O (FoxO) signaling pathway, and transcriptional misregulation in cancer, while the target genes of downregulated DEmiRNAs were mostly enriched in transforming growth factor-beta (TGF-beta) signaling pathway, proteoglycans in cancer, dorso-ventral axis formation, hepatitis B, and prolactin signaling pathway (Table 3).

**Table 3:**
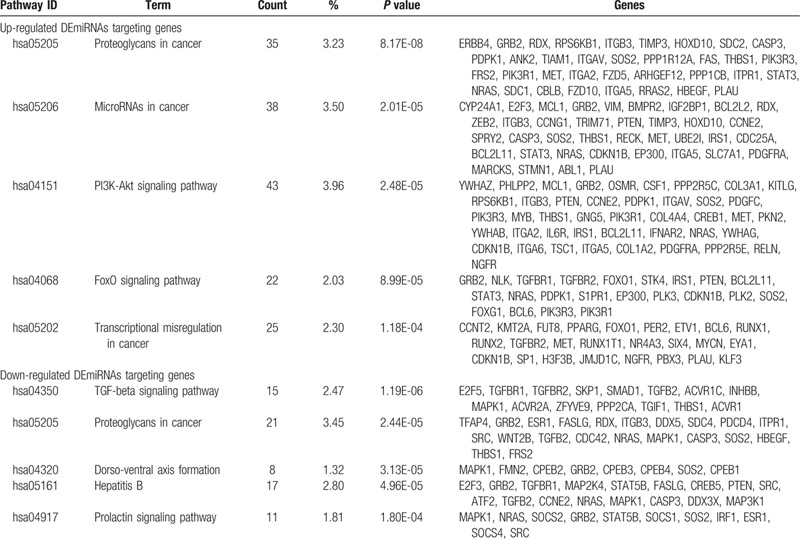
KEGG pathway analysis of DEmiRNAs target genes (Top 5).

### 3.5 PPI network and modules analysis

**Figure 1:**
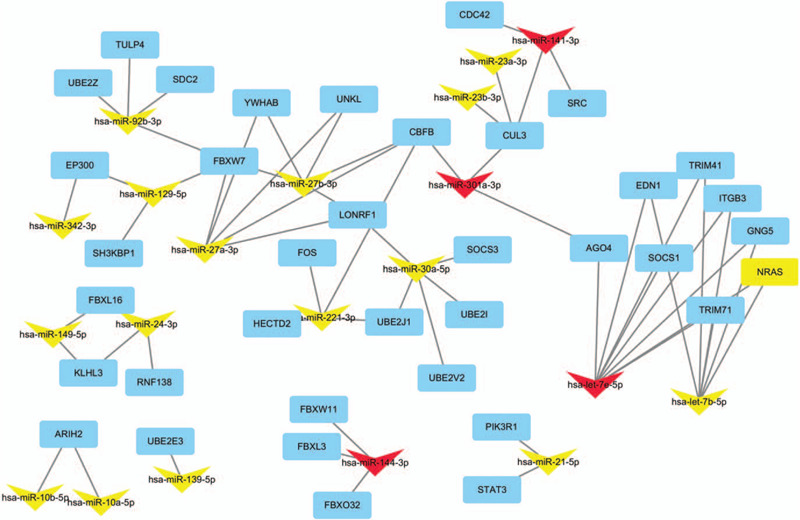
Regulatory network of the hub genes and DEmiRNAs that targeted them. The round rectangle nodes indicate hub gene ranked according to 11 ranked methods in Cytohubba. V-shape nodes represent serum exosomal DEmiRNAs. The yellow nodes stand for upregulated DEmiRNAs and red nodes for downregulated DEmiRNAs.

Protein interactions among the target genes of DEmiRNAs were analyzed by the STRING online database. Finally, a total of 1322 nodes and 3515 edges were identified from the PPI network program. Subsequently, the network was visualized in cytoscape and the top 3 significant modules were selected from the PPI network using MCODE (Fig. 1). The KEGG pathway enrichment analysis of the top 3 module genes were then performed. Genes in module A were mainly enriched in ubiquitin mediated proteolysis and protein processing in the endoplasmic reticulum. Genes in module B were mostly associated with spliceosome, mRNA surveillance pathway, and endocytosis, and PI3K-Akt signaling pathway. Finally, genes in module C were associated with the thyroid hormone signaling pathway, Huntington's disease, and notch signaling pathway (Table 4).

**Table 4:**
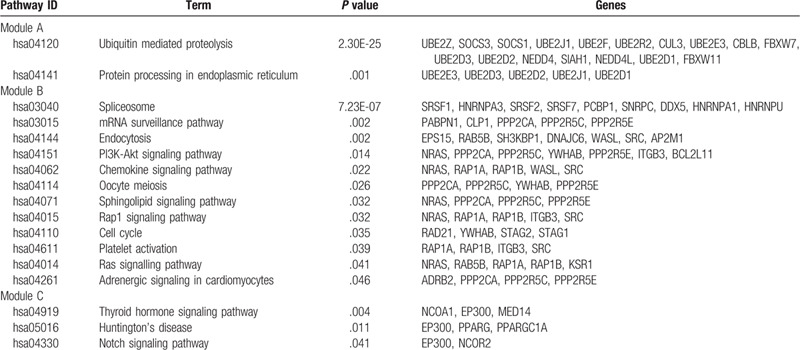
Functional and pathway enrichment analysis of the genes in the module.

### 3.6 Identification of hub genes and construction miRNA-mRNA network

In the present study, the top 10 hub genes were ranked according to 11 ranked methods in cytoHubba. Combining the results of 11 ranked methods of cytoHubba, 37 hub genes were determined for further analysis. Among them, 4 overlapping hub genes including phosphoinositide-3-kinase regulatory subunit 1 (PIK3R1), SRC, cell division cycle 42(CDC42), and E1A binding protein p300 (EP300) were identified according to 8 ranked methods (Betweenness, BottleNeck, Closeness, Degree, EcCentricity, MNC, Radiality, and Stress) in cytoHubba (Table 5). The 37 hub genes and 20 DEmiRNAs that target them were selected to construct miRNA-hub genes regulatory network in colorectal cancer (Fig. 2  ).

**Table 5:**
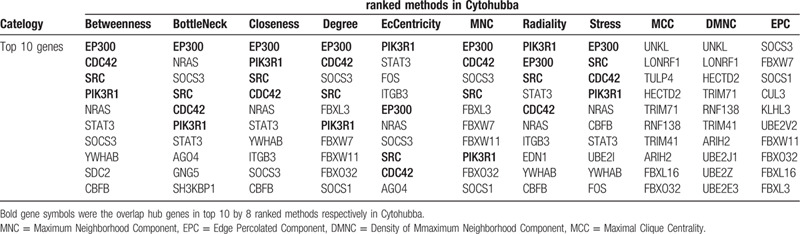
Hub genes ranked in cytoHubba.

**Figure 2:**
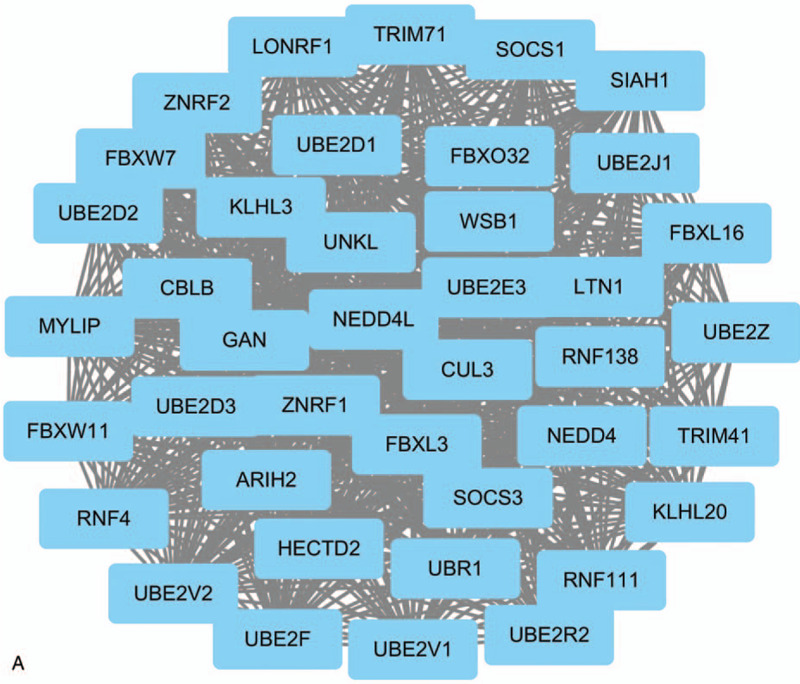
Top 3 modules identifed from the protein–protein interaction network. (A) Module A, (B) module B and (C) module C.

**Figure 2 (Continued):**
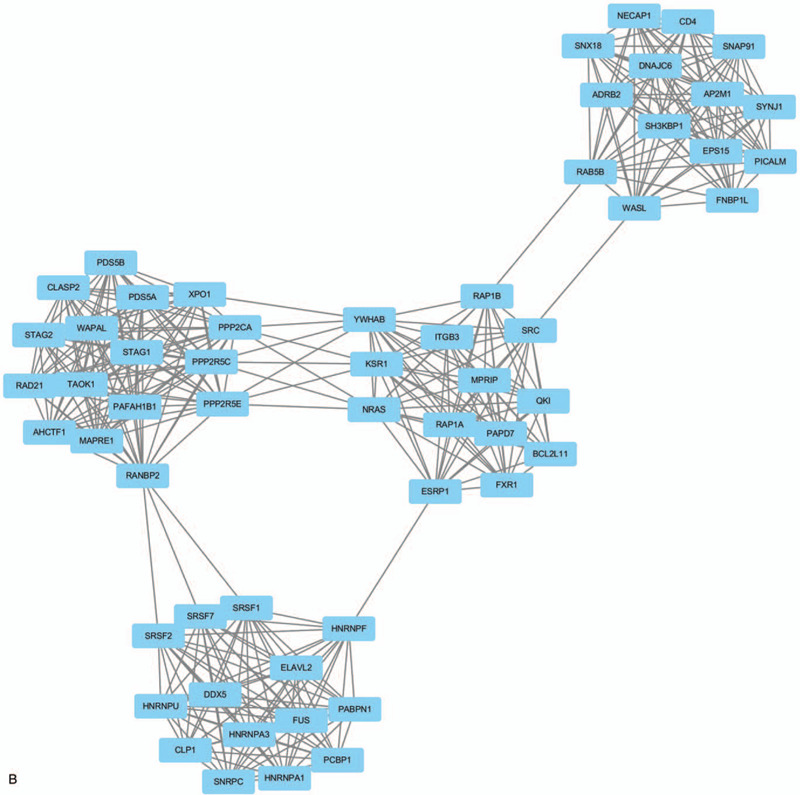
Top 3 modules identifed from the protein–protein interaction network. (A) Module A, (B) module B and (C) module C.

**Figure 2 (Continued):**
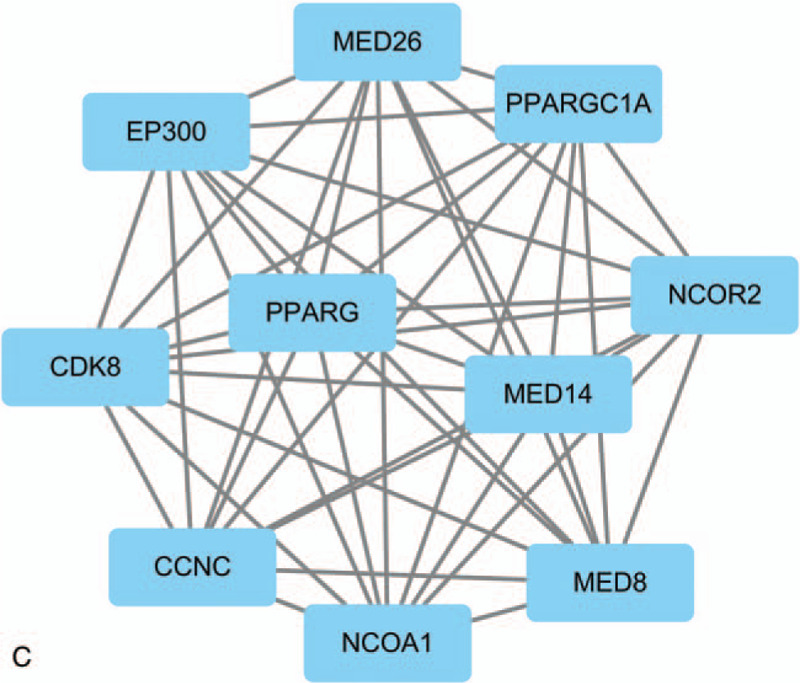
Top 3 modules identifed from the protein–protein interaction network. (A) Module A, (B) module B and (C) module C.

## 4 Discussion

Recent studies revealed serum exosomal miRNAs may play a critical role in cancer development and progression.^[15,23–25]^ Bioinformatics data-mining approach based on publicly available databases is a useful tool to reveal potential mechanisms and signaling pathways in various cancers.^[26–28]^ In the present research, DEmiRNAs were identified based on GEO databases and their target genes were predicted by multiple target predicting programs. The GO functional analysis indicated that the target genes of DEmiRNAs were mainly involved in transcriptional regulation, protein binding and transcription factor activity. The results were consistent with the knowledge that miRNA-dependent transcriptional regulation plays important roles in cancer development and progression^[29–32]^ and miRNA interacted with transcription factors to regulate the cancer-related genes in the pathogenesis of CRC.^[33–35]^

KEGG pathway enrichment analysis revealed that target genes of upregulated DEmiRNAs were mainly associated with proteoglycans in cancer, microRNAs in cancer, PI3K-Akt signaling pathway, FoxO signaling pathway, and transcriptional misregulation in cancer. During malignant transformation and tumor progression, the expression of proteoglycans (macromolecules essential for the tumor microenvironment) is significantly altered.^[36]^ Previous studies have shown that many proteoglycans are key molecular effectors of cell surface and pericellular microenvironments and contribute to diverse biological functions in various cancers including proliferation, adhesion, angiogenesis, modulating cancer progression, invasion and metastasis.^[37–39]^ Recent experimental evidence supported proteoglycans as miRNA targets involved in cell proliferation, apoptosis, adhesion, migration, and invasion in cancer progress.^[40,41]^ Studies have demonstrated upregulated miRNA 21 exerted its oncogenic activity by targeting the programmed cell death 4 (PDCD4) in CRC.^[42]^ PDCD4 plays a role in cell apoptosis as a tumor suppressor gene and encodes extracellular matrix proteoglycans which bind to the eukaryotic translation initiation factor 4A1 and inhibit its function by preventing RNA binding the tumor suppressor.^[43]^ In this study, we found serum exosomal miRNA 21 was upregulated and PDCD4 was a target gene of miRNA 21 in colorectal cancer. This finding was consistent with previous studies. The results suggested serum exosomal miRNA 21 may regulate cell progression by suppression of PDCD4 via proteoglycans in cancer pathway in CRC. In addition, the disturbance in PI3K-Akt and FoxO signaling pathway has been highly noted in CRC.^[44,45]^ Several studies show miRNAs including miR-125a,miR-135b,miR-182, miR-10b, and miR-21 affected colorectal cancer cell proliferation, migration, invasion, and pathological angiogenesis via PI3K-Akt pathway.^[46–49]^ Interestingly, this study also indicated miR-10b and miR-21 involved in colorectal cancer progress via the PI3K-Akt pathway. Therefore, serum exosomal miRNAs may play important roles in cancer progress via various pathways and help us to elucidate the potential mechanism of colorectal cancer metastasis.

The target genes of downregulated DEmiRNAs were mainly related to the TGF-beta signaling pathway, Proteoglycans in cancer, Dorso-ventral axis formation, Hepatitis B, and Prolactin signaling pathway. TGF-beta signaling is a key pathway in regulating cancer progression. Recent studies indicated several miRNAs affected cell proliferation, invasion, microenvironment modification by regulating the TGF-beta signaling pathway in CRC.^[50–52]^ Up-to-date, the role of pathway Dorso-ventral axis formation in cancer was unclear, but several studies have shown dorso-ventral axes regulated development of fetal and adult gastrointestinal structures and organs.^[53]^ Chronic hepatitis B infection has been associated with malignancy, most notably hepatocellular carcinoma. In recent years, the link between chronic hepatitis B infection and colorectal cancer has been reported. Many studies suggested hepatitis B infection increased the risk of colorectal liver metastasis and hepatitis B infection was associated with advanced colorectal adenoma development.^[54–56]^ Although the role of some signaling pathways remain unclear, they also provide us with some tips for understanding the mechanism of colon cancer progression

Based on the PPI network generated by target genes of DEmiRNAs, the most significant 3 modules were filtered from the PPI network complex. Module A analysis revealed ubiquitin mediated proteolysis and protein processing in endoplasmic reticulum were associated with the development of CRC. Ubiquitin mediated proteolysis system involved in a variety of basic cellular processes including cell cycle progression, cell proliferation, DNA replication, and apoptosis.^[57]^ The endoplasmic reticulum (ER) controls the biogenesis of nascent proteins entering the secretory pathway and also responds to the presence of misfolded proteins by targeting them for proteolysis via a process known as ER-associated degradation (ERAD). During ERAD, substrates are selected, modified with ubiquitin, removed from the ER, and then degraded by the cytoplasmic 26S proteasome.^[58]^ Increasing evidence indicates that the ubiquitin-proteasome system (UPS) plays an important role in various cancer. In this study, we found ubiquitin-conjugating enzyme E2 including ubiquitin conjugating enzyme E2 E3 (UBE2E3), ubiquitin conjugating enzyme E2 D3 (UBE2D3), ubiquitin conjugating enzyme E2 D2 (UBE2D2), ubiquitin conjugating enzyme E2 J1 (UBE2J1), and ubiquitin conjugating enzyme E2 D1 (UBE2D1) involved in the pathways of ubiquitin mediated proteolysis and protein processing in endoplasmic reticulum. The results indicated module A plays a role in colorectal cancer development via ubiquitin proteasome system. Module B analysis indicated multiple pathways were involved in colorectal cancer, and the majority of the pathways were associated with the ras family genes and the phosphatase 2A regulatory subunit B family gene. The results suggested the function of module B focused on the regulation of cell cycle, cell growth, and division in colorectal cancer.^[59,60]^ Module C was associated with the thyroid hormone signaling pathway, Huntingtons disease, and Notch signaling pathway. In the further functional analysis of genes in module C, we found the genes mainly focused on transcriptional regulation in pathway.^[61,62]^ So we speculated that the function of module C may be related to transcriptional regulation in the development of CRC.

We also identified hub genes using cytohubba in cytoscape software and 4 hub genes including PIK3R1, SRC, CDC42, and EP300 were listed by 8 topological algorithms. PI3K enzymes are a conserved family of lipid kinases that phosphorylate the inositol 3’-OH groups of membrane phosphoinositides.^[63]^ Class IA PI3K comprises a p110 catalytic subunit and a p85 regulatory subunit. PIK3R1 encodes the PI3K regulatory subunit p85α, while PIK3R2 and PIK3R3 encode p85β and p55γ respectively.^[64]^ P85 subunits regulate PI3K activation by modulating the stability, conformation, and localization of the catalytic subunit.^[65]^ P85α acts as a tumor suppressor, which strongly binding to the p110 catalytic subunit and increases its stability and inhibits its catalytic activity.^[66]^ PIK3R1 deletion in mouse led to a gradual change in hepatocyte morphology and finally induced hepatocellular carcinoma development.^[67]^ Previous study also showed PIK3R1 was significantly downregulated in early-stage colon adenocarcinoma group compared to normal group.^[68]^ In our study, PIK3R1 was identified as a target gene of miR-21–5p, while miR-21–5p significantly upregulated in serum exosomes of colorectal cancer compared to normal group. Therefore, we speculated that miR-21 may play a role in the carcinogenesis and development of colorectal cancer by inhibiting PIK3R1 gene. Similarly, a recent study indicated miR-21 knockdown suppresses cell growth, migration, and invasion partly by inhibiting PI3K/AKT activation via direct targeting PIK3R1 in breast cancer.^[69]^

Src as one of the most proto-oncogenes plays a crucial role in several malignancies and has become a key factor in colorectal cancer pathogenesis. Src is a non-receptor cytoplasmic tyrosine kinase which interacts with Receptor tyrosine kinases (RTKs), G protein-coupled receptors (GPCRs) and integrin receptors to facilitate the activation of signal transduction pathways such as the Ras-mitogen-activated protein kinase (Ras/MAPK), PI3K/Akt, Src/Focal adhesion kinase (FAK) complex signaling networks and signal transducer and activator of transcription (STAT) signaling pathways. The outcome of this crosstalk is the dysregulation of the tumors properties such as the increase in cell proliferation and survival, angiogenesis, tumor invasion, and metastasis.^[70,71]^ Previous research has shown that Src expression was increased in CRC samples compared with normal colonic mucosa and increased activity significantly correlated with stage.^[72]^ In recent years, studies have shown that miRNAs regulate cell proliferation, cell migration, and angiogenesis in colorectal cancer by targeting Src.^[73–75]^ In this study, miR-141 significantly reduced in serum exosomes of colorectal cancer and Src was predicted as a target gene of miR-141. Therefore, we speculated that miR-141 may play a role as a tumor suppressor via targeting Src gene in CRC. Previous study also has confirmed miR-141 inhibits proliferation and migration of colorectal cancer SW480 cells.^[76]^

Cdc42 may function as molecular switches and signals in multicellular pathways influencing various biological responses such as motility, morphology, and gene expression.^[77]^ Overexpression of cdc42 has also been found in many human cancers such as lung cancer,^[78]^ CRC,^[79]^ and breast cancer.^[80]^ Several studies indicated deregulation of cdc42 induced cellular transformation through disturbing the activity of Ras and epidermal growth factor receptor^[81]^ and promoted cell migration by mediating fibroblast growth factor and vascular endothelial grown factor.^[82]^ The results of our studies suggest that miR-141 may obstruct tumor growth and metastasis by targeting the cdc42 gene in CRC. Interestingly, consistent with our findings, miR-141 was demonstrated to suppress prostate cancer stem cells and metastasis by targeting cdc42 genes.^[83]^

EP300 functions as histone acetyltransferase to regulate transcription via chromatin remodeling and plays a role in the processes of cell proliferation and differentiation.^[84]^ EP300 is generally considered to be a classical tumor-suppressor gene. Critical tumorigenic pathways (including TGF-β, p53, and Rb) require EP300 cofactor activation to mediate the transcription of target genes.^[84]^ It is clear that EP300 functions as tumor suppressors in mice, which deficiency results in the development of hematological malignancies.^[85]^ EP300-deficient increased motility and invasive properties in breast cancer cells.^[86]^ Recent studies also show EP300 promotes cell differentiation and apoptosis and represses cell proliferation in colon cancer.^[87]^ The findings of our study indicated serum exosomal miR-342 may promote colorectal carcinogenesis by targeting EP300 gene. Similar to our research, miR-342 might promote lymph node metastasis in lung adenocarcinoma by targeting EP300.^[88]^

In the present study, several limitations should be mentioned. First, because the database of the serum exosomal miRNA profiles of colorectal cancer is rare, only a microarray profile can be analyzed. Second, our study is based only on computational analysis and lacked further experimental verification of exosomal miRNA functions and target genes.

In conclusion, the present study provides a comprehensive bioinformatics analysis of serum exosomal DEmiRNAs and their relationship with target genes in CRC. Our study provides a series of potential pathways and hub genes for future investigation into the function of serum exosomal miRNAs in the development of CRC. However, further experiments are required to validate these predictive results.

## Author contributions

HW and XC performed the analysis of the data. LB and XZ wrote the manuscript. XZ designed the study. All authors read and approved the manuscript.

## Supplementary Material

Supplemental Digital Content
